# Visualizing the Reading Activity of People Learning to Read

**DOI:** 10.16910/jemr.10.5.5

**Published:** 2017-11-15

**Authors:** Oleg Špakov, Harri Siirtola, Howell Istance, Räihä Kari-Jouko

**Affiliations:** Visual Interaction Research Group, TAUCHI Research Center University of Tampere, Finnland

**Keywords:** eye tracking, reading assessment, gaze visualization

## Abstract

Several popular visualizations of gaze data, such as scanpaths and heatmaps, can be used independently of the viewing task. For a specific task, such as reading, more informative visualizations can be created. We have developed several such techniques, some dynamic and some static, to communicate the reading activity of children to primary school teachers. The goal of the visualizations was to highlight the reading skills to a teacher with no background in the theory of eye movements or eye tracking technology. Evaluations of the techniques indicate that, as intended, they serve different purposes and were appreciated by the school teachers differently. Dynamic visualizations help to give the teachers a good understanding of how the individual students read. Static visualizations help in getting a simple overview of how the children read as a group and of their active vocabulary.

## Introduction

Imagine a class of schoolchildren developing their reading skills. They read the text silently, and they might indicate their progress by pointing to the current word with a finger. Their teacher moves around and observes, and intervenes when someone has problems. An experienced teacher knows the ones who really struggle and those who read fluently. However, there is usually a large number of pupils that are in between -- they do have occasional reading difficulties, but generally they advance steadily. For the teacher, it is a challenge to assess the progress of this group, or gain an overview of progress in the whole class.

The scenario above has many variations, but common to them all is that the teacher is getting insufficient information about individual readers. Eye tracking is a technology that reveals where a person is looking at when they use a computer, and it can be used, e.g., to provide detailed information on how reading progresses. Eye trackers are becoming cheaper and ubiquitous, and it is time to consider how they could be applied to analyzing the progress of reading skills.

We are developing a system for use in schools to analyze reading performance. Data from individual readers can be logged, so that a child's reading progress over time can be studied. If all of the children in a class are using the system, then the words causing problems to several children can be shared with the teacher as they happen. The teacher could use this information immediately to focus on these problems. Alternatively, the teacher could review the reading performance of some, or all, of the children after the lesson.

Eye tracking produces a large amount of data, so it is essential to find efficient visualizations for these needs. We first discuss the visualization of gaze data, and then introduce our system, a web application that has been developed to analyze users' reading performance. Finally, we describe how the system visualizes the collected data.

## Visualization of Gaze Data

Karn, Ellis, and Juliano [ 10 ] gave the following characterization for the data abstraction levels of gaze data, along with the most commonly used data types:

**First order data** Raw, unfiltered data:𝑥, 𝑦 position, pupil diameter, and blink signal. Some trackers give the distance (𝑧-coordinate), and some trackers do not output the unprocessed data at all.

**Second order data** Fixations, saccades, and pursuit eye movements (gaze smoothly following a target).

**Third order data** Scanpaths, fixation times for predefined areas of interest (AOIs), and matrix of transition probabilities between areas of interest.

**Fourth order data** Scanpath shape, complexity, and variability.

These data types are the building blocks for the visualizations of gaze data, and the most common visualizations are based on the second and third level data. The visualization of fourth order data is more demanding and usually requires considerable data aggregation (as demonstrated in the scanpath trend analysis by Eraslan, Yesilada, and Harper [ 6 ]). The visualization of gaze data has been studied extensively, and a recent review by Blascheck et al. [ 2 ] reports the state of the art. In this section, we review the concepts and methods relevant to the visualization of reading.

### Scanpath

**Figure 1:**
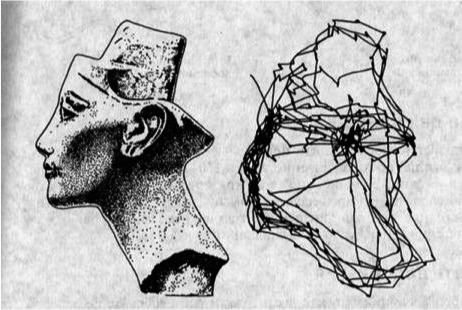
Scanpath of eye movements (on the right) during free examination of an image of the Egyptian Queen Nefertiri 25, p. 181.

A common static visualization of gaze data is a *scanpath*, a diagram of nodes and edges where the nodes are fixations and the edges are saccades. A scanpath is usually drawn as an overlay on top of the stimulus. In its most basic form, a scanpath is presented as any undirected graph, without decorating the nodes Figure 1.

**Figure 2:**
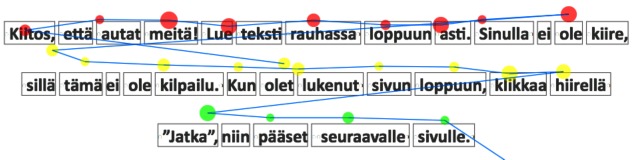
Scanpath with node size depicting the duration of fixation. The color of node indicates the associated text line.

The scanpath visualization has many useful variations. The nodes of a scanpath can be encoded to show the duration of a fixation, either by color, or more commonly, by size (Figure 2. This allows the viewer to reconstruct the events: the order and duration of fixations. The color of fixations has been used to encode the speed of the person’s gaze [ 11 ].

Scanpath with node size depicting the duration of fixation. The color of node indicates the associated text line.

**Figure 3:**
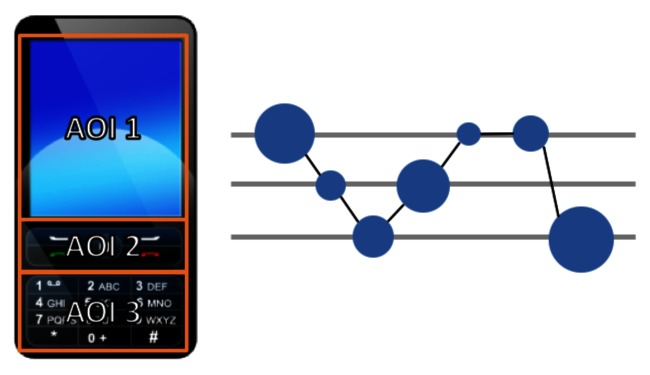
On the right, time plot of a scanpath where a person has viewed the image on the left (2). Horizontally the time, and vertically the AOIs in the same order as in the image. Used with permission.

As noted by Blascheck et al. [ 2 ], visual clutter is the primary problem of the scanpath visualization. If many nodes in a scanpath, or if several scanpaths are overlaid, the result is difficult to interpret. In addition, comparing the similarity of scanpaths is a challenge. The proposed solutions include the averaging or bundling of scanpaths to simplify the diagram, adjusting the alpha channel of the drawing, and inspecting the horizontal and vertical locations of fixations separately. Another approach in situations where AOIs that have a natural linear ordering is to emphasize the time aspect of a scanpath in a time plot Figure 3, having time and AOI as the axes of a scatterplot [ 15 ]. This presentation accentuates the pattern of scanpaths and allows easier comparisons.

### Heatmap

**Figure 4:**
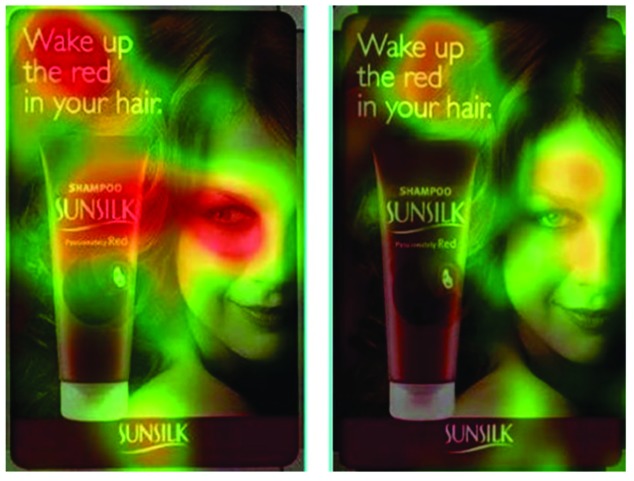
Heatmap: the product goes unnoticed if the model does not look at it (13).

Another common visualization of gaze data is a *heatmap*, which is based on statistical cluster heat maps [ 24 ] and fixation maps. In statistics, shaded matrix displays have been used for well over a century (see [ 12 ] for an example). In a fixation map, the stimulus being observed is overlaid with an opaque layer that becomes increasingly transparent on the areas most heavily observed [9, p. 44]. In a heatmap, the overlay layer is semitransparent, and the intensity of observation is encoded into color Figure 4.

The heatmap visualization is easy to implement and understand, and it scales remarkably well when a large number of observation sessions need to be aggregated. There are many variations of the technique, such as realtime heatmaps [ 5 ] and a variation where the eye gaze data modulates the transparency of the heatmaps [ 19 ]. The real-time heatmap is a rendering of heatmap on top of a video, with the help of a graphics processing unit (GPU).

## Visualization tools for analyzing gaze data in reading

**Figure 5:**
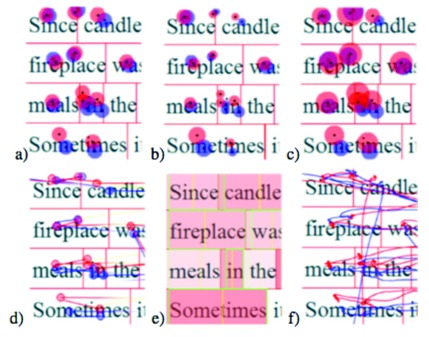
The six different data visualizations in EyeMap: a) fixation plot with fixed circle radius; b) fixation plot with fixation length encoded into circle size; c) pupil size encoded into circle size; d) gradually colored (encoding distance and direction) saccade lines with gaze points; e) word heatmap with word boundaries; and f) raw scan path. (21).

**Eyemap**. EyeMap is a freely available Flash-based eye movement data visualization tool targeted for reading research [ 21 ]. It requires a relatively spatially-accurate (less than 0.2°) tracker with a high sampling frequency (>250 Hz) to facilitate letter-level analysis. EyeMap provides six different visualizations of the gaze data Figure 5.

In conjunction with EyeMap, a novel XML format for gaze data was provided, specifically designed for reading research, to ease data import from a variety of eye trackers. Also yacc/lex-based data converters for the most common tracker formats were provided. EyeMap also supports the full Unicode standard, allowing it to be used with almost any language.

EyeMap allows integration of audio with the stimulus and visualization, facilitating the analysis of read-aloud studies. Adobe Flash was an unfortunate choice of platform, because it will be replaced with more secure and advanced technologies in the near future.

**Figure 6:**
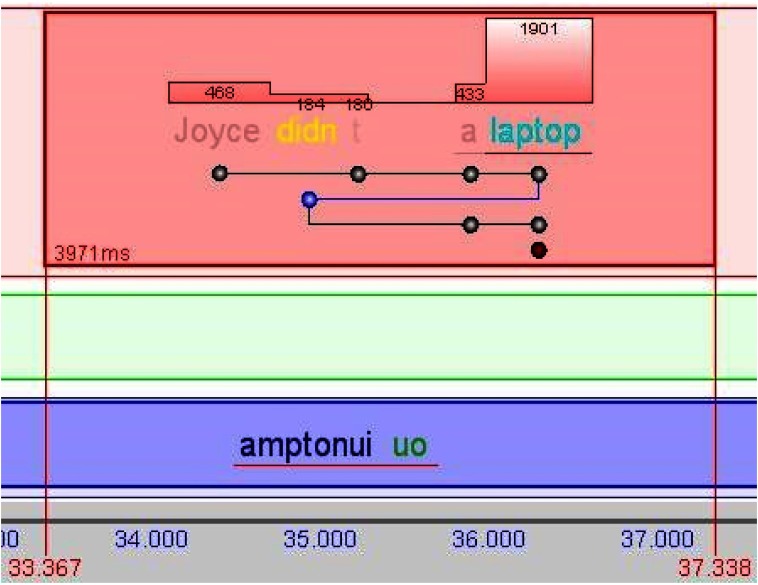
KiEV: visualization of reading the source text (top panel), reading the target text (middle panel, blank in this block), and typing the new translation (bottom panel) (20).

**KiEV**. Another visualization of reading data was developed in connection of translation studies [ 20 ]. It focuses on displaying reading and writing activities as blocks plotted against time. Each block represents an action continued without significant interruption Figure 6. Typing new text can overlap reading, but reading the source and target texts cannot take place at the same time.

Within reading blocks, all text read by a reader is displayed as well as the reading order. Regressions are emphasized with a distinct color. Bars above words represent fixations and their duration, thus reflecting the cumulative reading duration within the block. Most of the words are rendered with a transparency value that is inversely proportional to the accumulated reading time. The words starting or ending a regression are emphasized with distinct colors.

**SocialReading**. Cheng et al. [ 3 ] have built a system called SocialReading to improve users’ reading comprehension. The idea is to track expert users’ (e.g. teachers) gaze during reading, and then annotate the text with visualizations for less-experienced readers (e.g. students). The area of interest in SocialReading is a paragraph, and they use a number of intra-AOI and inter-AOI measures to create comprehension-improving visualizations. The intra-AOI measures include reading speed and reading count (for a paragraph), and the inter-AOI measure is switching frequency between AOIs. The reading speed is encoded in the value (level of gray, darker is faster) of an AOI. The number of times the text was read is indicated by the width of the paragraph border. The switching frequency between AOIs is encoded into line width that connects the two paragraphs Figure 7aFigure 7b.

**Figure 7a:**
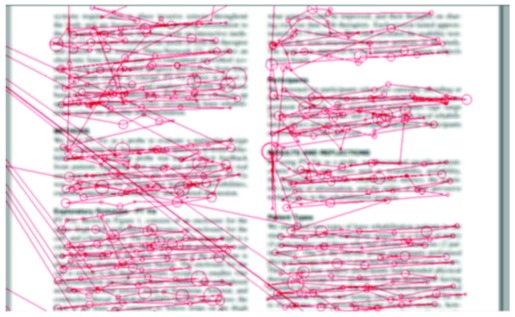
SocialReading: The raw data collected from expert readers (3).

**Figure 7b:**
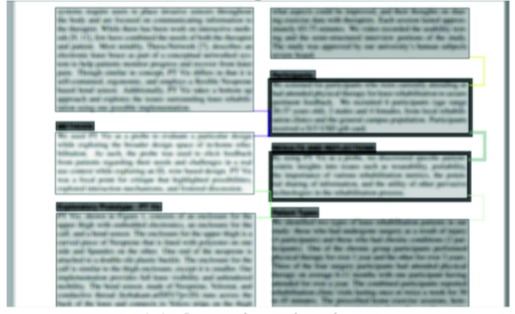
SocialReading: The visualizations of experts’ reading behaviour (3).

SocialReading has been evaluated with a user study. In the study, two faculty members read a paper, and the paper was annotated according to their gaze data. Two student groups then read the paper, one group with annotations and another without them. The outcome was that gaze-based annotations (or visualizations) resulted in better comprehension, and increased the similarity in the reading process between experts and non-experts.

**iTraceVis**. Clark and Sharif [ 4 ] have developed a component for iTrace, called iTraceVis, that creates visualizations of how programmers read source code. iTrace automatically maps gaze position to source code elements, and makes it possible for iTraceVis to visualize how programmers try to understand large software systems.

### Summary

The four visualizations of reading behavior reviewed here are quite different, and reflect assumptions made about the needs of the respective target user groups. As the user group and their tasks are able to be more precisely defined, the higher the level of abstraction becomes and the further away the visualization is from the raw gaze data. The most precise user group description is possible with iTraceVis, then KiEV, then SocialReading, and the least with EyeMap.

## System description

We have developed an open source web application 1 that shows the text to be read in a browser. It tracks the reader’s eye movements, and streams the collected gaze data up to a cloud database for further analysis. In addition, our system has a game that is really an eye tracker calibration in disguise. The gamified calibration – and the verification of the calibration – are essential in the school environment, because the teacher does not have time to oversee these activities individually for each student. This section describes the details of the system.

### User interface

**Figure 8:**
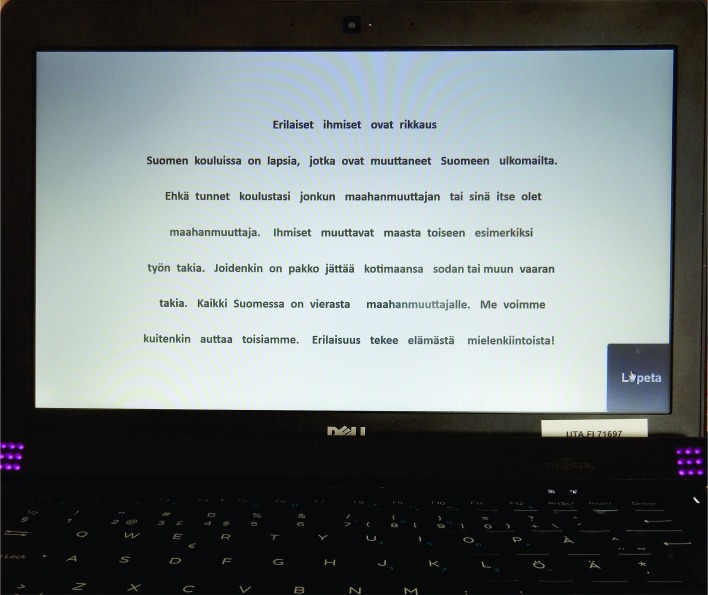
User interface. The page with the text fills the 13" screen in full-screen mode

From the users’ perspective, the system is just an ordinary web page with text to be read ( Figure 8. While reading, there is no indication that the eye movements are being observed. The text is centered, both horizontally and vertically, because the eye tracker accuracy tends to deteriorate towards the peripheral screen area. This is not completely natural, and may affect the reading process as the length of line, font size and typeface, and the contrast between text and background all have influence 8. However, this limitation is always present in onscreen reading.

### Hardware

Currently, the system uses myGaze eye trackers manufactured by Visual Interaction GmbH 2, in conjunction with SensoMotoric Instruments 3. The choice of the model was based on several factors, such as price, lack of usage limitations (SDK license), mobility, and a comparison of device performance. The device is capable to provide 30 gaze points per second with an average accuracy of under 30 pixels in all directions. This accuracy was observed about 15 minutes after calibration, in the class setting, where students were gazing at 48×24 pixels targets distributed across the screen.

The eye trackers were attached to Dell laptops with metal strips glued slightly below a 13" screen. Students were sitting approximately 50 cm from the screen. The screen resolution was set to 1366 × 768 pixels; thus, one visual angle degree corresponded to about 35 pixels. The laptops were powerful enough to run myGaze software smoothly in parallel with the web application for text presentation and data collection in Firefox browser (Intel i5-5300U@2.3GHz, 8MB).

### Software

*ETU-Driver*^4^ with a myGaze plug-in and activated WebSocket server was used to stream gaze data from the tracker to the web application. The web application utilized the *GazeTargets* library to handle gaze data received over WebSocket. This library mapped gaze points onto text words and notified the application when a word got or lost the focus. It also computed fixations in realtime using the dispersion-based algorithm described in [ 16 ]. Word focusing events were then directed to two modules, one for syllabification of the words under certain conditions, and one for tracking the history of words visited by gaze. The latter module also received fixations. When reading/tracking was finished, this module uploaded the setup and recorded data (lists of text words, syllabified words, and fixations) into a Firebase database.

### Gamified calibration

There were two main reasons for developing a gamelike calibration for this study: 1) students would be able to perform the calibration without help from the teacher or study supervisor; and 2) it would motivate students to complete the calibration carefully on repeated occasions.

**Figure 9:**
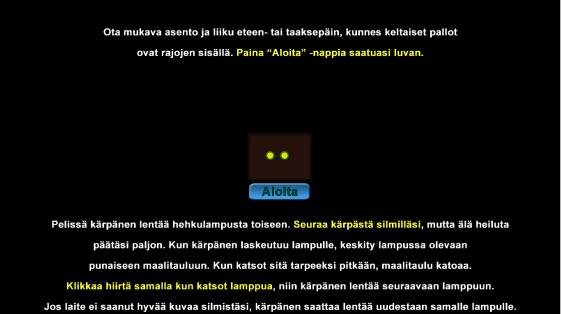
The calibration start screen

**Figure 10:**
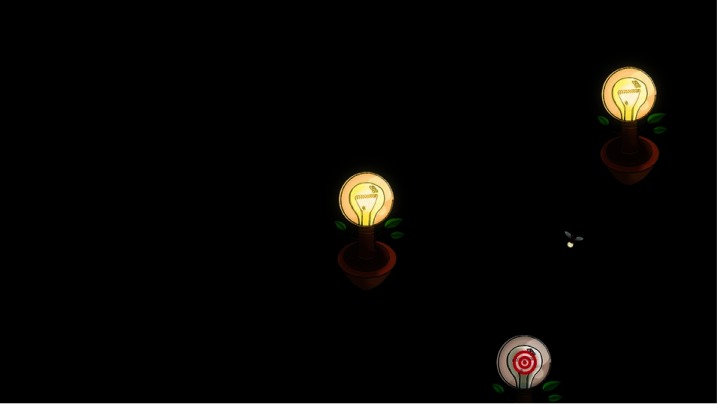
Calibration display: two lamps switched on, a firefly, and the lamp switched off with the bull-eye. Three of the five targets are visible at this point of the calibration.

**Figure 11:**
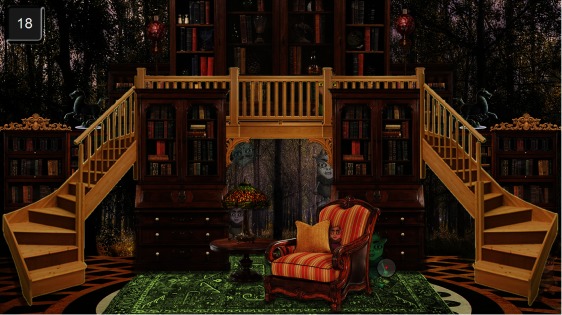
Calibration game: a student should point at the creatures with gaze (one can be seen behind the chair leg) and click mouse or press space-bar to “catch” them.

The initial screen displayed locations of both eyes in the tracker camera view and an instruction of how to choose the best sitting position, descriptions of what will be displayed next, and actions needed to complete the calibration Figure 9. The button Start (Aloita) was enabled only if both eyes were visible and tracker-to-eyes distance was between 40 and 65 centimeters. After a student clicked the button (or pressed the space bar), the first target appeared in the center of a blank screen. The target was shown as a bullseye attached to a lamp that was switched off. An animated firefly moved from the top onto this lamp Figure 10. After a second, the lamp was switched on. A student then clicked the mouse or pressed the space bar to display the next lamp and move the firefly onto it. There were five calibration points (lamps) altogether. The firefly might revisit a lamp if the corresponding calibration point was considered as not calibrated reliably. At the end of the calibration the lamps disappeared and an image with ten embedded, partly visible, dimmed creatures appeared. The students were required to find the creatures, look at them, and click the mouse or press the space bar to “catch” them Figure 11within 30 seconds. The pointer position was controlled by gaze and this acted as a calibration check. Players got points for each creature caught and, if all creatures were caught before time was up, for each second saved

### Syllabification

The chosen means of supporting readers automatically when difficulties reading a particular word were detected was *syllabification*, or displaying the word with its component syllables separated by hyphens. Finnish is a syllable-stressed language with transparent orthography. Therefore the role of syllables is heavily emphasized in Finnish reading instruction [ 7 ].

Word syllabification for Finnish follows some simple rules^5^ that are easy to implement. Still, there are exceptional words as well as some borrowed or compound words for which the syllabification does not follow the rules. Syllabified representations for all such words found in the text used in this study were hard-coded.

**Figure 12:**
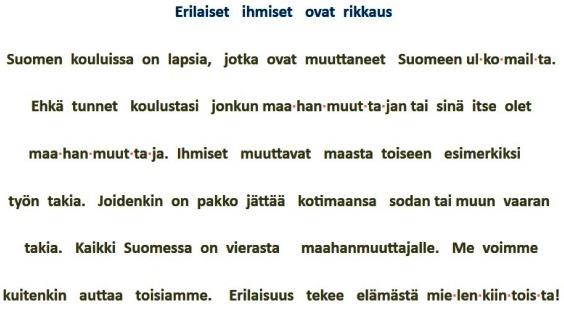
Text page with four words syllabified with short red hyphen.

The criterion for the syllabification of a word to be displayed was simply the cumulative time spent reading it: as soon as the cumulative reading time of a word exceeded a certain threshold, the word was automatically replaced with its syllabified representation. Since the hyphens used to separate syllables took up a certain space, words in the text were separated by a slightly larger space than usual. Short red hyphens were used to separate syllables, as shown in Figure 12.

To accommodate differences in reading speed between students and to ensure that syllabification occurred while the reader was still looking at the word, we developed a method to estimate the best trigger threshold. For this, we displayed an introductory page before the actual text. The application collected word reading events as students read this page, and calculated the threshold based on average word reading duration when they moved on to the first page of the task text. The threshold was set at 4 times the average word reading duration, but limited to at least 1.5 seconds and at most 3 seconds.

## Visualization of reading data

In the visualization of reading, we abstract the gaze data one step further from fixations and saccades: we want to see how long and how many times the *words* are fixated, and in which order. Often the gaze data is analyzed by dividing the gaze target into areas of interest (AOIs) – in our case, the words constitute the AOIs.

We have implemented five different methods to present the gaze data of reading. Two of them – *Gaze replay* and *Word replay* – are animated views to show the change over time, and *Gaze plot, Word reading durations* and *Student summary *are static views that summarize the data. In this section we describe the visualizations.

### Gaze plot

**Figure 13:**
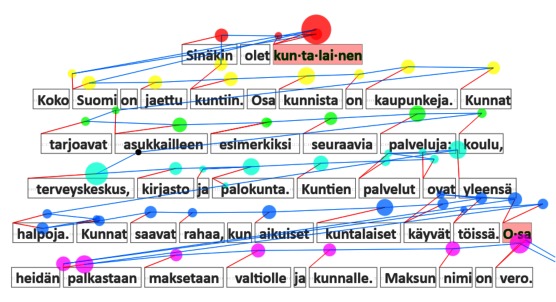
Gaze plot: fixations as circles and saccades as blue connector lines. The color-coding indicates association with text lines, and the red lines map each fixation to the corresponding word.

*Gaze plot* is perhaps the most traditional visual presentation of gaze data, akin to plots used by Yarbus [ 25 ]. *Gaze plot* is a visualization of a single reader’s fixations and saccades overlaid on the text Figure 13. The fixations are shown as semi-transparent circles, and the circle’s size (area) is proportional to the duration of the fixation. The saccades are shown as blue lines connecting the fixations. The color coding of the fixation circles maps them to the lines of the text, and the unmapped fixation circles are drawn in black. The fairly complex mapping algorithm is described in an article under review [ 18 ]. The gaze-to-line mapping is similar to the one suggested by Beymer and Russell [ 1 ].

This kind of visualization reveals some aspects of reading behavior, such as backtracking, which is generally an indication of problems, either in reading or comprehension. It also reveals if any of the words were syllabified during the reading (as “kun-ta-lai-nen” is in Figure 13). In addition, *Gaze plot* serves as a diagnostic tool showing the quality of tracking and the gaze-to-word mapping.

### Gaze replay

**Figure 14:**
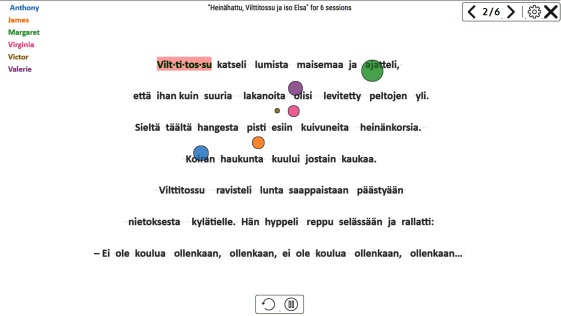
Gaze replay. The gaze pointer color corresponds to the color of student name. Words that were syllabified for any student are shown with pink background.

As the name suggests, *Gaze replay* is an animation of gaze behavior in a reading session. It shows the gaze pointer as a circle having the size in proportion to the current fixation duration – so the size of the circle keeps growing while the gaze remains at the same location. Figure 14 shows a moment in the animation where six readers’ gaze pointers are displayed. The readers and the pointers are connected by color-coding, which is shown in the top left corner. The replay is synchronized to make all readers start at the same time, and a check mark appears next to a reader’s name whose data has finished to replay.

Syllabified words are displayed with a pink background at the (relative) time of the syllabification event, thus at the end of the replay all the syllabified words for all the readers are shown. The replay can be paused or restarted, and there will be a timeline slider in the forthcoming version.

*Gaze replay* allows the observation of reading as it would happen in real time, either for a single reader or a group of readers. It is easy to see if there are problems in reading strategy or behavior, and to make comparisons.

*Gaze replay* can be seen as an animated version of the *Gaze plot* visualization. The occlusion and clutter problem is circumvented by showing the saccades as movement only, without leaving a trail behind. This approach allows the observation and comparison of gaze behavior of several readers at the same time.

### Word replay

**Figure 15:**
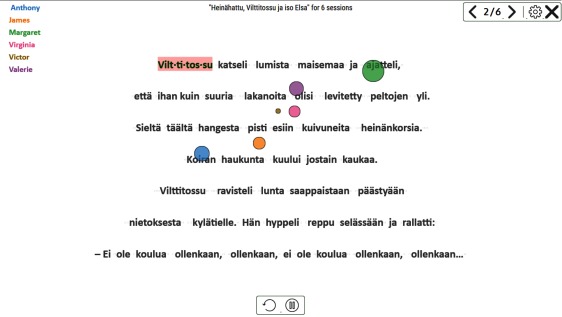
Word replay. The list of words and students’ replay tracks are displayed vertically. Cell darkness is proportional to the word reading duration by the corresponding student.

*Word replay* is another animated view of the gaze data from a reading session. This visualization presents the progress of reading as a grid where we have readers in the columns and the text on the rows, one word per row Figure 15. Each reader’s gaze pointer is indicated as a constant size orange circle that moves down the column as reading progresses. The gaze pointer is visible only when a fixation on a word is detected.

**Figure 16:**
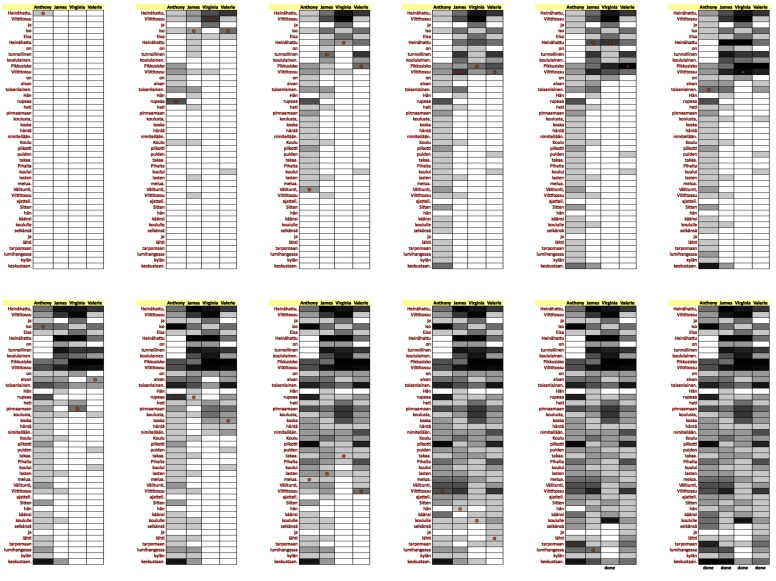
Progress of the Word Replay visualization.

At the beginning of *Word replay* all the cells in the grid are white. The gray level of cells gets gradually darker based on the cumulative gaze time spent on the corresponding word. There are 20 levels of gray, each corresponding to 250 milliseconds of gaze time. Finally, a cell turns black if the accumulated gaze time exceeds five seconds. When the reader has finished reading, the text “done” is displayed at the bottom of the corresponding column Figure 16.

The *Word replay* visualization shows the progress of reading over time, and indicates how the reading time is distributed over the text. This visualization provides insight on several things, such as: • Who are the slowest/fastest readers? • Which words are difficult for some/all readers? • Are there differences in reading strategies? • Does anyone exhibit re-reads (backtracks)? • Do grammatical cases cause slowdowns?

In the snapshot in Figure 15, it is clear that Irene and Sharon are taking much longer reading the text while the rest of the readers are progressing fluently. John has skipped reading the title (the first three words) and also skipped some small elements (the m-dash and the word “niin”). In addition, all but one of the fast readers have a slight slowdown at the word “ikkunasta”, possibly caused by the grammatical case (elative case of the word “ikkuna”); for Steven, the color of that cell is the same as for the preceding cells, so that word did not cause a slowdown in his reading.

## Word reading durations

**Figure 17:**
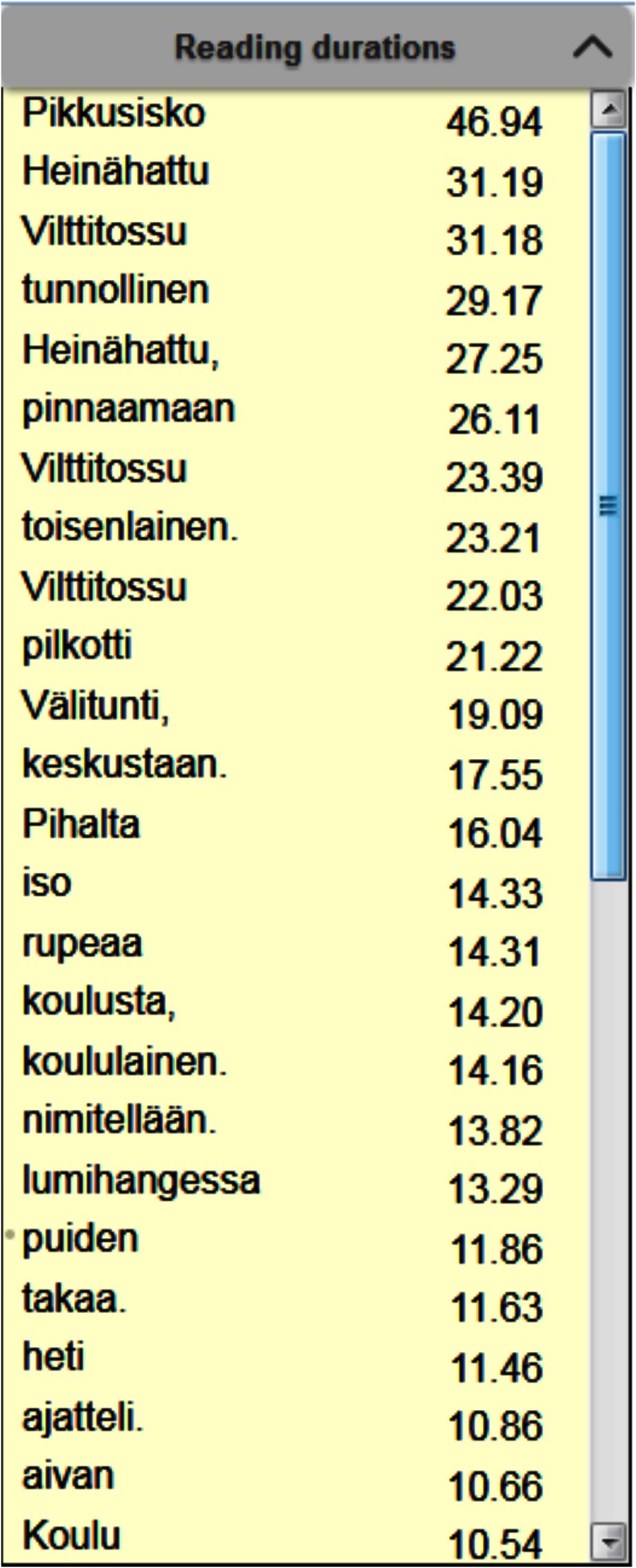
Table showing the sum of word reading durations for each word in descending order, in seconds. The table is generated for currently selected readers.

It is also possible to view the reading durations of words in a tabular form Figure 17. The contents of this table are shown for the currently selected readers, and it displays the sum of reading durations for each word, in descending order. This table gives a quick insight of which words were the most difficult for the readers. Generally, the top of the list is occupied by longer and less common words.

## Student summary

**Figure 18:**
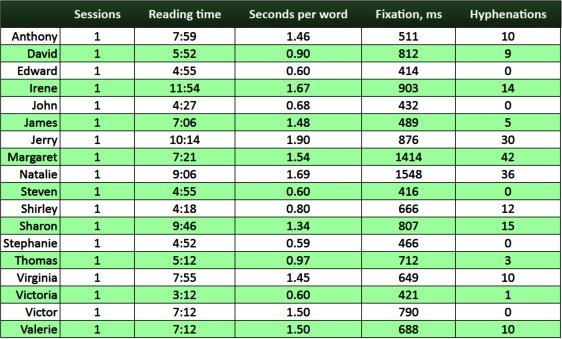
Student summary with statistics for each student on the number of texts read, total time spent on reading, average time spent on reading a word, average fixation duration and total number of syllabified words

Finally, *Student summary* is an interactive table showing a summary of the reading performance for each reader Figure 18.. The statistics include the number of reading sessions, the overall time spent on reading, the average time to read a word, the average fixation duration, and the overall number of syllabified words. The user can sort the table by columns.

This visualization provides a quick quantification of the readers’ performance. However, the current version does not support the viewing of progress over time (several reading sessions).

## Assessment of the Visualizations

The visualizations were assessed by means of a questionnaire to collect opinions from experienced teachers. Two of the respondents were from a local school where the system was initially tested, and all the rest were participants of the Interactive Technology in Education [ 9 ] conference where the project organized a hands-on ‘Future Lab’ to demonstrate the system.

### Demography

Twenty respondents (twelve women and eight men) from 28 to 60 years of age (mean 46 years) agreed to fill in the questionnaire after they had tried out the system. Their teaching experience ranged from 2 to 36 years, 15 years was the average. Their teaching positions varied from primary school to university, including special needs education, vocational school and teacher training school. The subjects taught in the primary and secondary schools varied a lot, but most teachers were involved in teaching either L1 or L2 languages (the child’s native language or second language).

Four of the respondents did not provide a ranking for the visualizations, therefore the ranking summary is based on the answers of 16 participants.

### Results

In the first part of the questionnaire the respondents were asked to rate each visualization on a seven-point scale, ranging from *not at all informative* to *extremely informative*. In addition, they were asked to state one positive and one negative feature of the visualization.

**Figure 19:**
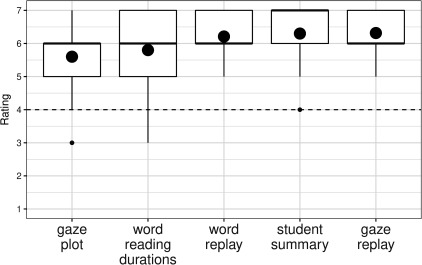
Rating of visualizations as boxplots with scale 1 to 7 (1 being not informative at all and 7 being extremely informative). The (large) black dot indicates the overall mean per visualization.

Figure 19shows the summary of the given ratings as boxplots. Overall, *Student summary* was rated *extremely informative* (median 7), and for all the others the rating was *very informative *(median 6).

**Figure 20:**
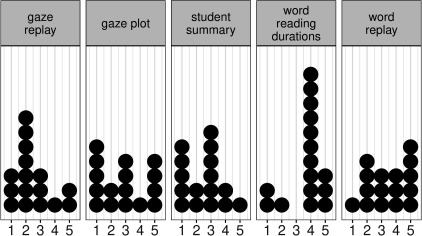
Ranking of visualizations as a dotplot with 1 being the most useful and 5 being the least useful.

In the second part of the questionnaire the respondents were asked to rank the visualizations in order of their usefulness Figure 20.. This result is slightly different from the rating result. *Student summary* and *Gaze plot* both received five first place rankings as the most useful for a teacher of students learning to read. If first place and second place rankings are taking into account, *Gaze replay* was the favorite because of many second place rankings. *Word reading durations* was mainly ranked fourth. The *Word replay* got fairly even ranking for all positions except for the ‘most useful visualization’.

## Discussion

In discussions after the questionnaires had been completed, the teachers pointed out that all visualizations were informative, so they found it hard to fill in the ranking table. They described *Gaze plot* as providing a good overview of data collected and revealing strategies that students used to read. Some noticed that the color-coding of fixations was useful and helped them in inspection of the gaze path, but some disagreed with this point of view, as the mapping was not accurate for some fixations.

*Word reading durations* helped teachers to quickly capture the words most difficult to read. *Gaze replay* gave an insight into “the general situation in the classroom in terms of reading ability”. Teachers reported that with *Gaze replay* it was easy to check whether the students’ reading behavior was as expected, whether the reading speed was sufficient, which words caused problems and what the differences were in the reading process between the readers. The gaze pointer color-coding was also thought to be clear.

Similar responses were recorded for the *Word replay* visualization. Teachers emphasized that persistent visualization of the accumulated reading time was helpful for identifying the difficult words quickly. It was noted that this identification was still possible after the replay had finished. Some, however, pointed out that the visualization was a little difficult to understand and it did not allow inspection of when readers jumped to the next line.

In *Student summary* teachers found that the reading data was presented clearly. They liked the ability to compare the reading speed and commented that even in the current implementation this summary would allow them to inspect students’ reading performance progress. They claimed they would use it when creating reports on student development. Some teachers wished to join this data with data collected in other assessments of student performance into a single table for convenience.

As a summary, *Gaze replay* and *Student summary* did well in both types of rankings. This was also confirmed in a more formal analysis, e.g., using the voting algorithm for finding the “winner” (the method with the fewest number of first place rankings is dropped, the other votes are adjusted accordingly, and the process is repeated). These two visualizations arrived at a tie for first place. However, the most important observations were that all visualizations had their own specific uses, and that people had different preferences. Since there is no need to exclude any of them, we will continue to provide all visualizations in future versions.

For many visualizations the teachers reported that they missed some kind of the text comprehension evaluation, therefore gaze data alone are not sufficient. According to the comments, reading speed would not say much about reading ability if text comprehension was insufficient. Reading comprehension assurance was the most often noted missing feature.

## Case Studies

Of particular interest to us were the teachers of the two classes whose students we tested. The teachers knew the children and anticipated each to show a certain level of reading proficiency. Students used the system in class. Six students read a text from screen, while the rest of the students read the same text from a textbook. No comprehension questions were asked, but sometimes the teacher selected some words for discussion after everybody in the class had finished reading.

**Figure 21:**
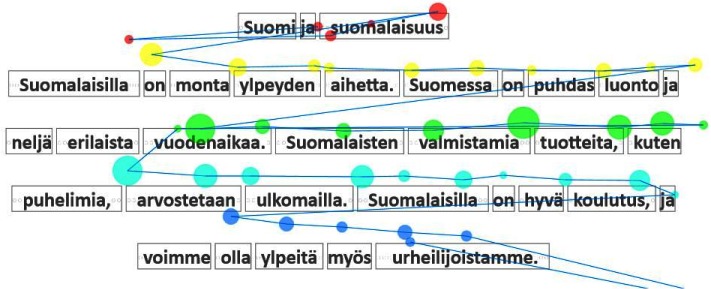
Gaze plot for Isabel.

**Figure 22:**
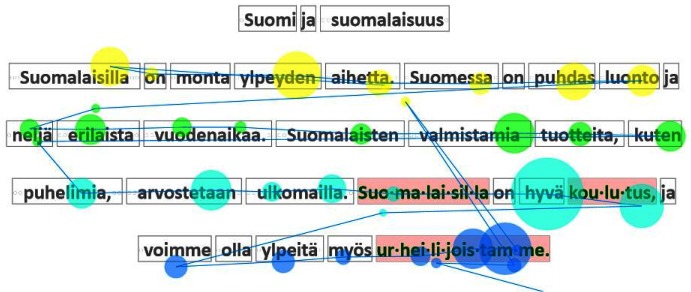
Gaze plot for Nancy.

Interestingly, there were surprises to both teachers. When we viewed the visualizations in a debriefing after collecting the data, both found in their classes one child that was a slower reader than they expected, and vice versa, one child that read faster than expected. For instance, Isabel 6 from the third grade participated in a session where six students read a three-page document. She finished second on all three pages, which was a surprise to the teacher. Her reading ability was obvious in, e.g., *Gaze plot* Figure 21.: the reading proceeds smoothly without getting stuck at any point. In contrast, Nancy was the slowest reader in the group of six. Her *Gaze plot* Figure 22. also shows progress at a regular, but much slower pace, mainly because of the longer fixations. This has led to three words being hyphenated on this page.

Figure 22 also illustrates a phenomenon that came as a surprise to us: many children skipped the title (first line) of the page. This initially had a significant negative impact on the gaze-to-word mapping, since it was based on the assumption that the children would read the texts linearly, from the first to the last line. When this behavior was discovered, the mapping was modified so that it now tries to match the data in two ways, namely, with or without the reading of the title line. shows that the algorithm has detected that the title line was not read.

We reviewed the visualizations one-by-one with the teachers. The third-grade teacher commented that *Gaze plot* was useful in showing whether reading progressed 6 All names have been changed. systematically, and whether a child had problems in understanding the text. This became evident if gaze jumped back and forth between lines. *Word reading durations* were useful for pinpointing unknown words that did not yet belong to the vocabulary of the children. *Gaze replay* was appreciated for showing the progress of many readers at the same time; this saved both time and helped to pick out the students that needed more attention. While *Gaze replay* thus was useful for sorting out the good readers from the less good ones, *Word replay* conversely was useful for picking out words that typically were skipped, or that took a long time. Finally, *Student summary* had many advantages: it could be used in formal evaluation, in planning support activities, and in communicating progress over time to parents.

In the debriefing sessions it became evident that teachers need some coaching in interpreting the gaze data, in particular to take into account that there is noise in the gaze points delivered by the tracker. This is caused by several reasons, including poor calibration and lack of concentration by the student. Consequently, the relative order of the gaze points and fixations is more important than their exact location. Nevertheless, both teachers were highly appreciative of being able to see the reading process “through the eyes of the student”.

## Conclusions

We have developed several new visualizations of gaze data collected from primary school students. The visualizations were devised for showing the reading proficiency of children who have fairly recently learned to read fluently, and with high variation in their reading skills.

The visualizations were evaluated using questionnaires filled in by twenty teachers. In addition, visualizations of data collected from the students of two classes, second and third grades, were analyzed in detail with the teachers.

Both evaluations highlight that the various visualizations have their place and serve different purposes. The dynamic visualizations (*Gaze replay* and *Word replay*) help to give the teachers a good understanding of how the individual students read. In addition, they help in comparing the skills of a group of students reading the same text. The static visualizations (*Gaze plot, Word reading durations, Student summary*) help in providing a simple overview of both the children and their active vocabulary. Several teachers explicitly commented that we should retain all the visualizations in future versions of our tool.

So far we have focused on developing visualizations of a single reading session. In future work we will also develop visualizations for showing the development of the reading skill of a student over several reading sessions.

## Acknowledgements

We are sincerely grateful to the children of the 2B and 3B classes and their teachers, Sanna Salonen and MarjaLeena Ämmänkoski, at Lamminpää Primary School for their cheerful and committed co-operation. Inka Hyrskykari was instrumental in running the data collection sessions at the school. Tiia Viitanen and Terhikki Kataja from Tampere University of Applied Sciences designed and produced the calibration game. Daniel Trifonov from Visual Interaction helped us with the myGaze software. Matias Vakkilainen from Sanoma Pro provided access to the reading material used in the case study.

*This research was funded by the Academy of Finland, project Private and Shared Gaze: Enablers, Applications, Experiences (GaSP)*^7^.

In presenting the questionnaire data we used *Statistical System R* [ 14 ] and *tidyverse packages* [ 23 ], especially *ggplot2* [ 22 ].

## Footnotes

https://uta-gasp.github.io

http://www.mygaze.com

https://www.smivision.com

http://www.sis.uta.fi/~csolsp/downloads.php

An example of rules can be found at https://web.stanford.edu/~laurik/fsmbook/exercises/FinnishSyllabification.html

All names have been changed.

ttp://gasp.sis.uta.fih
